# Identification of key genes associated with atrial fibrillation and hypoxia using WGCNA and machine learning technology

**DOI:** 10.3389/fcvm.2025.1614979

**Published:** 2025-11-28

**Authors:** Chao Wang, Mardan Muradil, Jianbin Huang, Jie Cai, Fangbao Ding, Li Zhang, Mengda Li, Chenglai Fu, Ju Mei, Zhaolei Jiang

**Affiliations:** 1 Department of Cardiothoracic Surgery, Xinhua Hospital, School of Medicine, Shanghai Jiaotong University, Shanghai, China; 2 Spine Center, Xinhua Hospital Affiliated to Shanghai Jiaotong University School of Medicine, Shanghai, China; 3 Neurological Surgery, Henan Provincial People’s Hospital, Henan, Zhengzhou, China; 4 Institute for Developmental and Regenerative Cardiovascular Medicine, Xinhua Hospital, Shanghai Jiao Tong University School of Medicine, Shanghai, China

**Keywords:** atrial fibrillation, hypoxia, weighted gene co-expression network analysis (WGCNA), machine learning, hub genes

## Abstract

**Background:**

Atrial fibrillation (AF) is among the most prevalent cardiac arrhythmias worldwide, and its incidence is steadily rising due to global aging. Hypoxia, a well-recognized trigger of AF, plays a pivotal role in the onset and progression of AF. However, the molecular mechanisms underlying the interplay between AF and hypoxia remain unclear, and specific biomarkers for this condition are lacking. This study aimed to identify key hypoxia-related genes associated with AF through an integrated bioinformatics approach that combines weighted gene co-expression network analysis (WGCNA) with machine learning (ML) algorithms, and to assess their potential diagnostic significance.

**Methods:**

This study employed an integrative approach combining weighted gene co-expression network analysis (WGCNA) and machine learning (ML) to identify key genes associated with AF under hypoxic conditions. AF-related gene expression data were sourced from the Gene Expression Omnibus (GEO) database, and hypoxia-related gene sets from the Molecular Signatures Database (MSigDB) database. WGCNA was employed to identify gene modules associated with AF, which were then intersected with hypoxia-related genes. Candidate hub genes were identified using random forest and least absolute shrinkage and selection operator regression. Their diagnostic performance was evaluated using receiver operating characteristic (ROC) curve analysis. A predictive nomogram was developed, and immune infiltration analysis and gene set enrichment analysis (GSEA) were performed to explore associated biological pathways and alterations in the immune landscape.

**Results:**

WGCNA identified 34 gene modules, with the most AF-relevant module comprising 624 genes. Intersection analysis and ML algorithms identified SLC6A6, BGN, and PFKP as key genes. ROC analysis demonstrated strong diagnostic potential. Immune cell profiling showed increased infiltration of M2 macrophages and dendritic cells in AF samples, with significant correlations to the expression of these hub genes.

**Conclusion:**

This study identified SLC6A6, BGN, and PFKP as key genes associated with AF under hypoxic conditions and successfully developed a diagnostic model with promising clinical applicability. These genes likely play important roles in hypoxia-mediated AF pathogenesis and are closely associated with immune cell infiltration, providing potential biomarkers for early diagnosis and precision treatment of AF. This study provides novel insights into the molecular mechanisms underlying the interplay between hypoxia and AF.

## 1 Introduction

Atrial fibrillation (AF) is the most prevalent cardiac arrhythmia worldwide, with an increasing incidence due to the progressive aging of the population. An epidemiological study in the United States estimated that the number of individuals with AF ranges from 3 to 6 million, and this figure is projected to rise to approximately 6 to 16 million by 2050 (1). Hypoxia is one of the common triggering factors of AF and serves as a critical driver of its sustained progression. The most frequently encountered conditions leading to hypoxia include coronary artery disease (2, 3) and obstructive sleep apnea syndrome (4). Multiple mechanisms underlie the induction of AF under hypoxic conditions, primarily involving atrial electrophysiological and structural remodeling, inflammatory responses, and oxidative stress (2, 5). Although extensive clinical and fundamental research has demonstrated a close association between AF and hypoxia, a thorough investigation into the molecular mechanisms concerning AF onset in a hypoxic state remains insufficient, particularly regarding the identification of definitive biomarkers. In recent years, weighted gene co-expression network analysis (WGCNA) has become an effective bioinformatics approach for finding key gene modules related to specific diseases based on gene expression data (6). Additionally, machine learning (ML) techniques like random forest (RF) and least absolute shrinkage and selection operator (LASSO) regression, have been widely applied in gene selection and disease prediction (7, 8). By integrating WGCNA with ML approaches, it is possible to identify critical genes related to AF and hypoxia with greater precision. Therefore, this study aimed to elucidate the molecular mechanisms linking hypoxia signaling with AF, identify key hub genes, and construct a diagnostic model based on these genes. Our findings will offer new perspectives and lay the theoretical groundwork for unveiling the pathogenesis of AF in hypoxic states and developing innovative diagnostic tools.

## 2 Methods

### 2.1 Data collection and preprocessing

**Figure 1:**
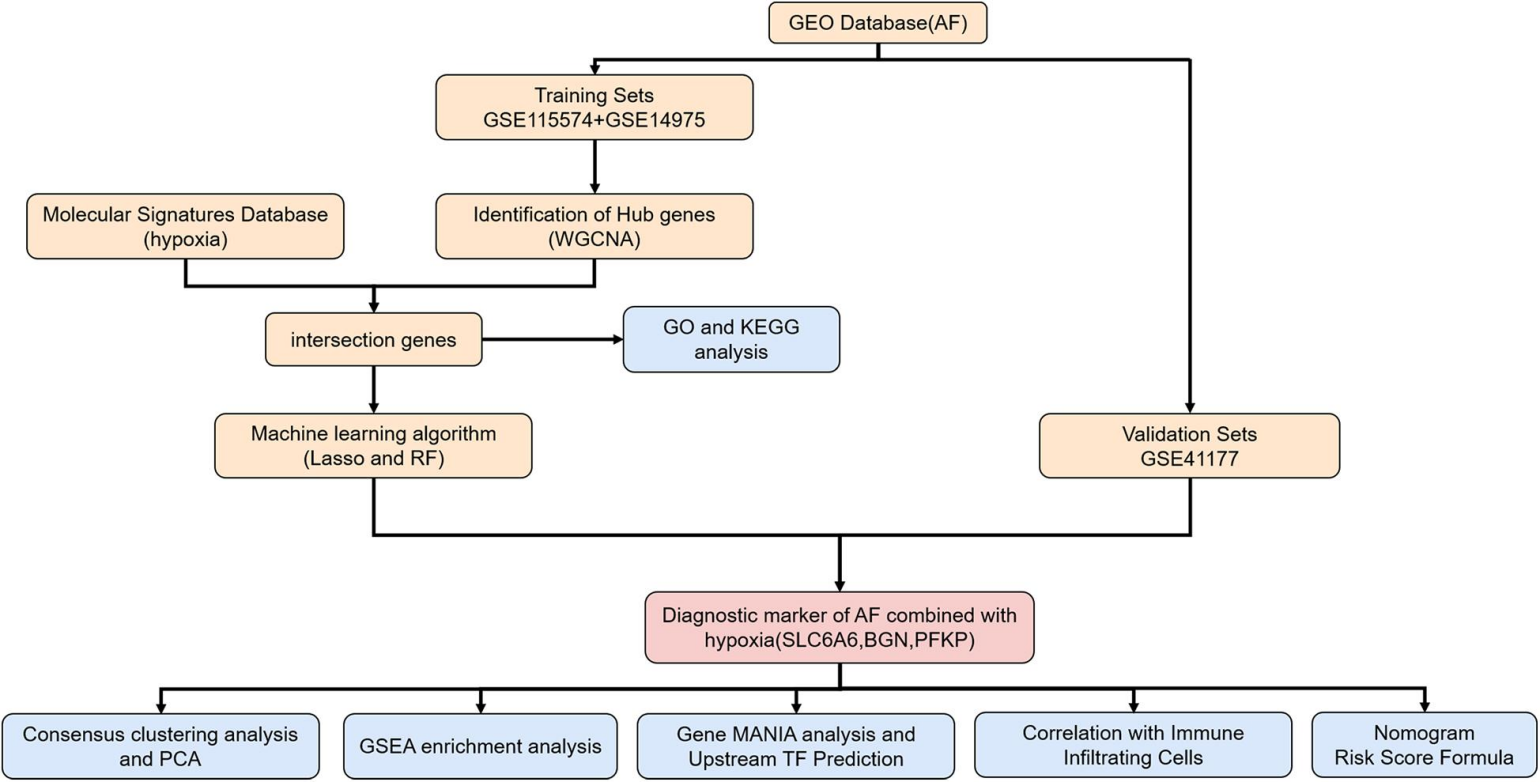
Flowchart of the study. AF, atrial fibrillation; WGCNA, weighted gene Co-expression network analysis; GO, gene ontology; KEGG, Kyoto encyclopedia of genes and genomes; LASSO, least absolute shrinkage and selection operator; RF, random forest; PCA, principal component analysis; GSEA, gene set enrichment analysis gene; MANIA, gene multiple association network integration algorithm.

The gene expression profiling data for AF were sourced from the Gene Expression Omnibus (GEO) (https://www.ncbi.nlm.nih.gov/geo/), with details presented in Table 1. The GSE115574 dataset comprises 59 samples, including 31 left atrial appendage (LAA) tissues from AF patients and 28 control tissues from people with sinus rhythm (SR). The GSE14975 dataset encompasses 10 samples, including 5 AF LAA tissues and 5 SR control samples. The GSE41177 dataset includes 38 samples, comprising 32 AF LAA tissues and 6 SR control samples. The gene-related information for all AF datasets was derived from the GPL570 platform. The GSE115574 and GSE14975 datasets were combined to create a training set, while GSE41177 was designated as an external validation set. After the raw dataset files were downloaded, genes with zero or negative expression values were excluded before log₂ transformation. Low-expression probes (mean log₂ intensity < 1 or detected in <50% of samples) and low-variance genes [median absolute deviation (MAD) < 0.15] were removed. A total of 21,653 genes meeting these criteria were retained for subsequent analyses. Expression values for each gene were then normalized to ensure their independence, facilitating subsequent computational processing. Raw expression data were retrieved using R 4.4.1 and the GEOquery package. Subsequently, an expression matrix was constructed and probes were mapped to their corresponding gene symbols. Duplicate genes and missing values were eliminated. If a probe corresponded to multiple genes, that particular gene was excluded to ensure data integrity. The final gene matrix was integrated, and the batch effect was corrected utilizing the ComBat function from the SVA package 3.52.0. Information on hypoxia-upregulated genes was sourced from the Molecular Signatures Database (MSigDB) (http://www.gsea-msigdb.org). Specifically, 200 hypoxia-related genes were obtained by querying the database using the “HALLMARK_HYPOXIA” keyword (Figure 1).

**Table 1:** Brief description of hypoxia and AF source dataset.

Disease/Pathological state	Data chip	Sample size		Data source	Year
		Normal control	Disease		
AF	GSE115574	28	31	Gene expression data from human left and right atrial tissues in patients with degenerative MR in SR and AF	2021
AF	GSE14975	5	5	Transcriptional profiling of left atrial myocardium from AF and SR patients	2019
AF	GSE41177	6	32	Region-specific gene expression profiles in left atria of patients with valvular atrial fibrillation	2019
Hypoxia	M5891	200		Genes up-regulated in response to low oxygen levels (hypoxia).	2015

AF, atrial fibrillation; SR, sinus rhythm.

### 2.2 WGCNA

To ensure the accuracy of WGCNA, the integrated gene matrix was further filtered, and genes expressed in over 50% of the samples were retained. The WGCNA package 1.73 was employed to assess the quality of samples and genes, ensuring that the data matrix was suitable for WGCNA. Sample hierarchical clustering was performed on the integrated gene-sample data to identify potential outliers (Figure 3A). A biologically significant scale-free network was developed utilizing the soft-thresholding parameter (*β*) as per the scale-free topology requirement. The “pickSoftThreshold” function was adopted for computing and selecting an appropriate *β* value, ensuring that the scale-free topology model fitting index (sftr squared) was approximately 0.8, thereby guaranteeing network robustness and biological relevance. Based on network topology analysis, a CutHeight value (height threshold) greater than 0.8 was selected for gene module construction, with at least 50 module genes. The gene module most strongly linked to AF was identified. Gene modules were defined via a topological overlap matrix (TOM) in combination with the dynamic tree-cut method. After module delineation, the eigengene was calculated for every module (module eigengene, ME). Finally, Pearson correlation coefficients were employed to evaluate the link of modules to clinical traits. The module most strongly related to clinical characteristics was identified as the key module and visualized within the trait-gene network.

### 2.3 Functional enrichment analysis of hypoxia- and AF-associated Hub genes

Our study identified the intersection between hypoxia-related genes and the most AF-relevant module genes from WGCNA. Their involvement in biological processes (BP), molecular functions (MF), and cellular components (CC) was explored through Gene Ontology (GO) analysis on the intersecting genes. Additionally, the Kyoto Encyclopedia of Genes and Genomes (KEGG) pathway analysis was conducted to characterize and describe gene functions. To ascertain the statistical enrichment of genes within KEGG and GO pathways, the ClusterProfiler package 4.12.6 was utilized. Pathways containing three or more significantly enriched genes with *p* < 0.05 suggesting significance.

### 2.4 ML-based key genes identification

Two commonly used ML algorithms: RF and LASSO, were utilized following the identification of hypoxia-AF intersecting genes. LASSO regression analysis was enabled by the “glmnet” package 4.1–8, while the RF model was generated via the “randomForest” package 4.7–1.2. Genes overlapping between the RF and LASSO analyses were considered potential hypoxia-associated AF biomarkers. Furthermore, to evaluate the diagnostic value, receiver operating characteristic (ROC) curves comprehensively present the model's classification performance at different thresholds.

### 2.5 Construction and evaluation of the predictive model

A nomogram model was built on the identified key gene set. Every risk factor was assigned a corresponding score, with the total score mapped to AF occurrence probability. Calibration curves reflected the concordance between observed and predicted results. The net benefit of the model in forecasting AF occurrence was examined through decision curve analysis (DCA).

### 2.6 Relationship between key genes and infiltrating immune cells

To investigate immune cell infiltration within the samples, this study utilized the CIBERSORT package 0.1.0, which employs the LM22 immune cell-specific gene matrix (LM22 signature) for correlation analysis. CIBERSORT was used to estimate the proportion of immune cells in each sample, and the results were integrated with sample classification data. Bar plots presented overall immune cell infiltration, and box plots illustrated differences across AF and normal samples. To explore the potential relationships of key genes with immune cells, the links of selected genes to each immune cell type were unraveled via Spearman correlation analysis. The obtained correlation coefficients and significance values were visualized in a heatmap. Data visualization was performed using the R packages “reshape” 0.8.9, “tidyverse” 2.0.0, “ggplot2” 3.5.1, and “pheatmap” 1.0.12. *p* < 0.05 denoted statistical significance.

### 2.7 Gene set enrichment analysis (GSEA) and prediction of upstream transcription factors

GSEA was employed to interpret the biological significance of specific genes in biological processes or diseases via the GSVA package 2.0.5 in R, with visualization implemented via the “ggplot2” 3.5.1 and “enrichplot” 1.24.4 packages. Gene sets meeting the adjusted *p* < 0.05 threshold were significant. A co-expression gene network was formed via GeneMANIA (http://www.genemania.org). NetworkAnalyst (https://www.networkanalyst.ca) helped to analyze the link of key genes to their associated transcription factors, facilitating the prediction of upstream transcriptional regulators.

### 2.8 Consensus clustering analysis and principal component analysis (PCA)

Clustering analysis algorithms were leveraged for validating and confirming the biological significance and effectiveness of identified key genes. Based on hypoxia- and AF-associated key genes, unsupervised consensus clustering was performed through the “ConsensusClusterPlus” package 1.70.0. AF patient subtypes were defined across all AF samples, with 50 iterations conducted to assess result stability. Key operational parameters were an 80% item resampling rate and a maximum k-value of 10. PCA was utilized to examine the differentiation among clustering groupings and to corroborate the clustering outcomes.

## 3 Results

### 3.1 Construction and processing of the AF dataset

**Figure 2:**
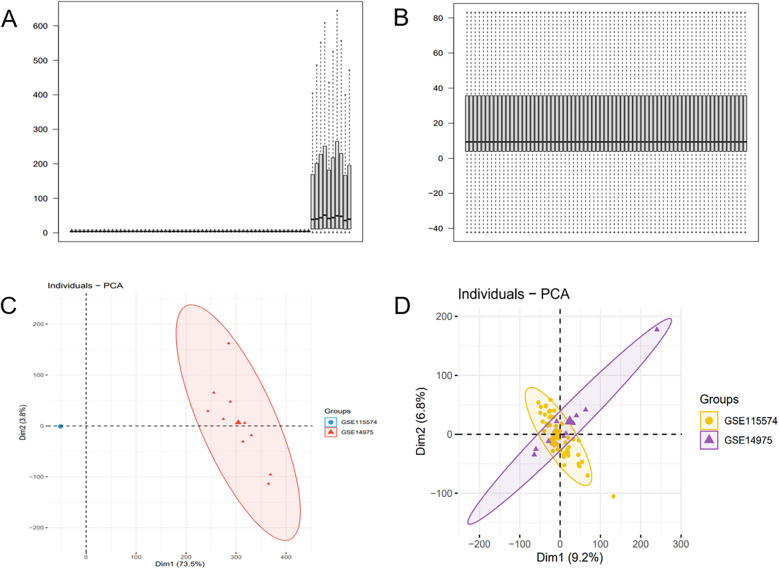
Batch effect correction results. **(A)** Boxplot of the AF dataset before batch effect correction. **(B)** Boxplot of the AF dataset after batch effect correction. **(C)** PCA of the AF dataset before batch effect correction. **(D)** PCA of the AF dataset after batch effect correction.

The batch effect was corrected on the GSE115574 and GSE14975 datasets (Figures 2A,B), comprising 69 samples, including 33 AF samples and 36 SR samples. 21,653 genes were identified, and PCA was undertaken to detect differences before and after correction (Figures 2C,D).

### 3.2 Identification of AF-associated genes using WGCNA

**Figure 3:**
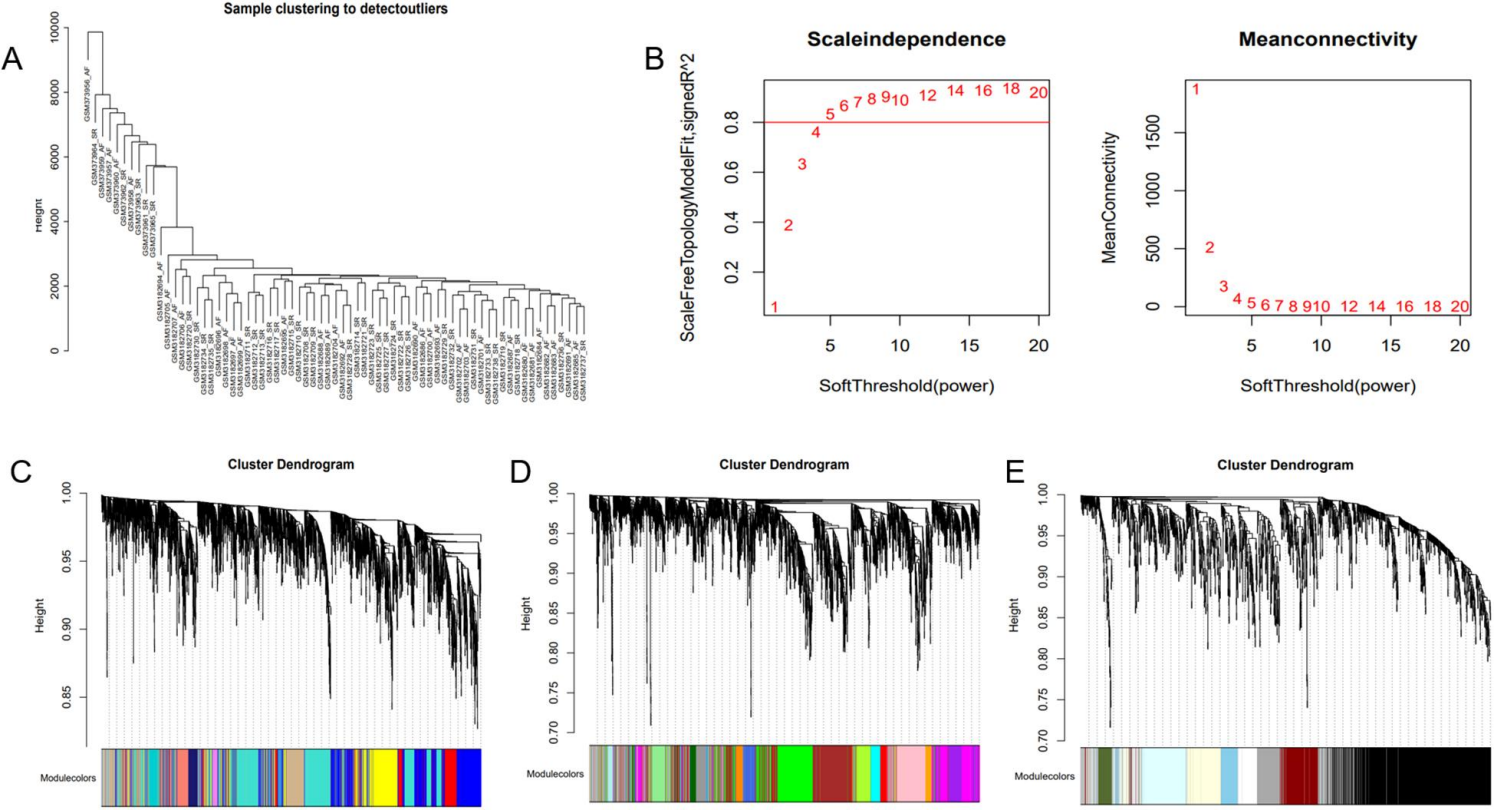
WGCNA. **(A)** Sample clustering dendrogram of AF. **(B)** Relationship of the fitting index with soft threshold (left) and the relationship of mean connectivity with soft threshold (right). **(C–E)** Module clustering dendrogram of the AF co-expression network with different colors representing different modules.

**Figure 4:**
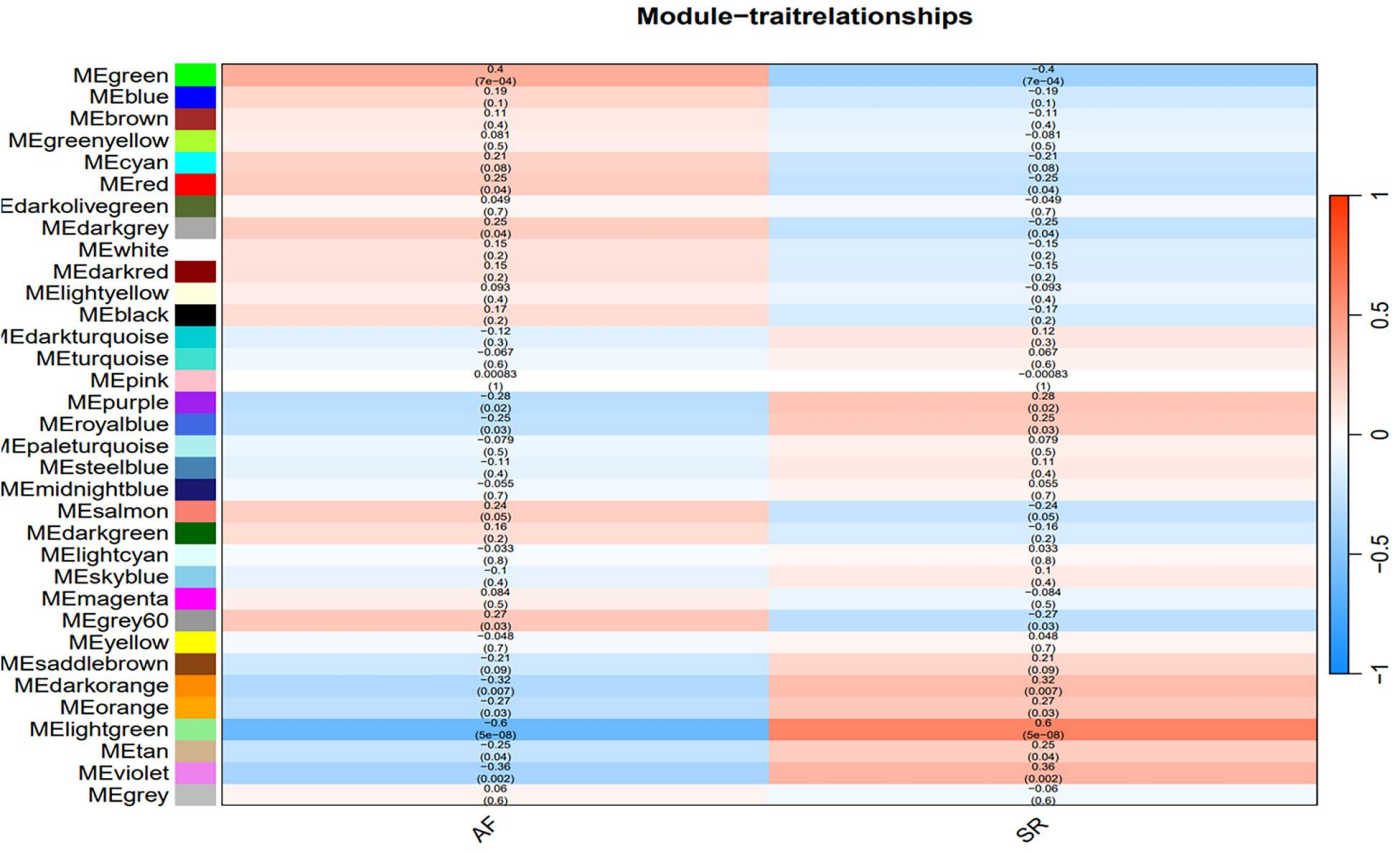
Heatmap of the correlation between gene modules and clinical characteristics of AF. Red indicates a positive correlation, blue indicates a negative correlation.

**Figure 5:**
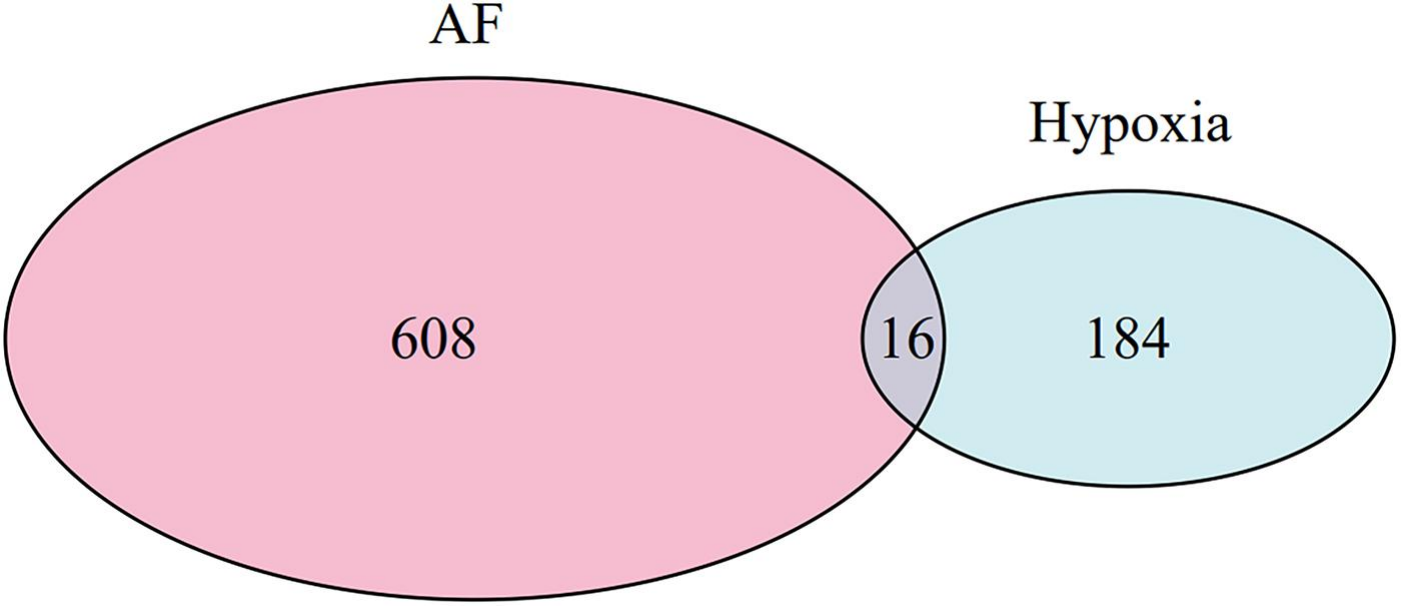
Venn diagram of AF-related genes identified by WGCNA and hypoxia-related genes.

The optimal soft-threshold (*β*) was determined as per the scale-free topology criterion. When the soft-threshold was 5, *R*^2^ exceeded 0.8 (Figure 3B). Gene clustering resulted in 34 modules, with the module dendrogram illustrated in Figures 3C–E. A heatmap illustrates the correlation of gene modules with AF-related clinical characteristics (Figure 4). Notably, the green module showed the highest relation to AF (*r* = 0.4, *P* = 7e-04), encompassing 624 genes. Intersecting the genes from the key AF-related module with the hypoxia gene set yielded 16 overlapping genes (Figure 5). An overlap of 16 genes was observed between the AF-associated “green module” (*n* = 624) and the HALLMARK_HYPOXIA gene set (*K* = 200), which was significantly greater than expected by chance (expected 5.76; hypergeometric *p* = 0.00056; OR = 2.56, 95% CI: 2–11), indicating a non-random enrichment of hypoxia-responsive genes within the AF-associated module.

### 3.3 Enrichment analysis

**Figure 6:**
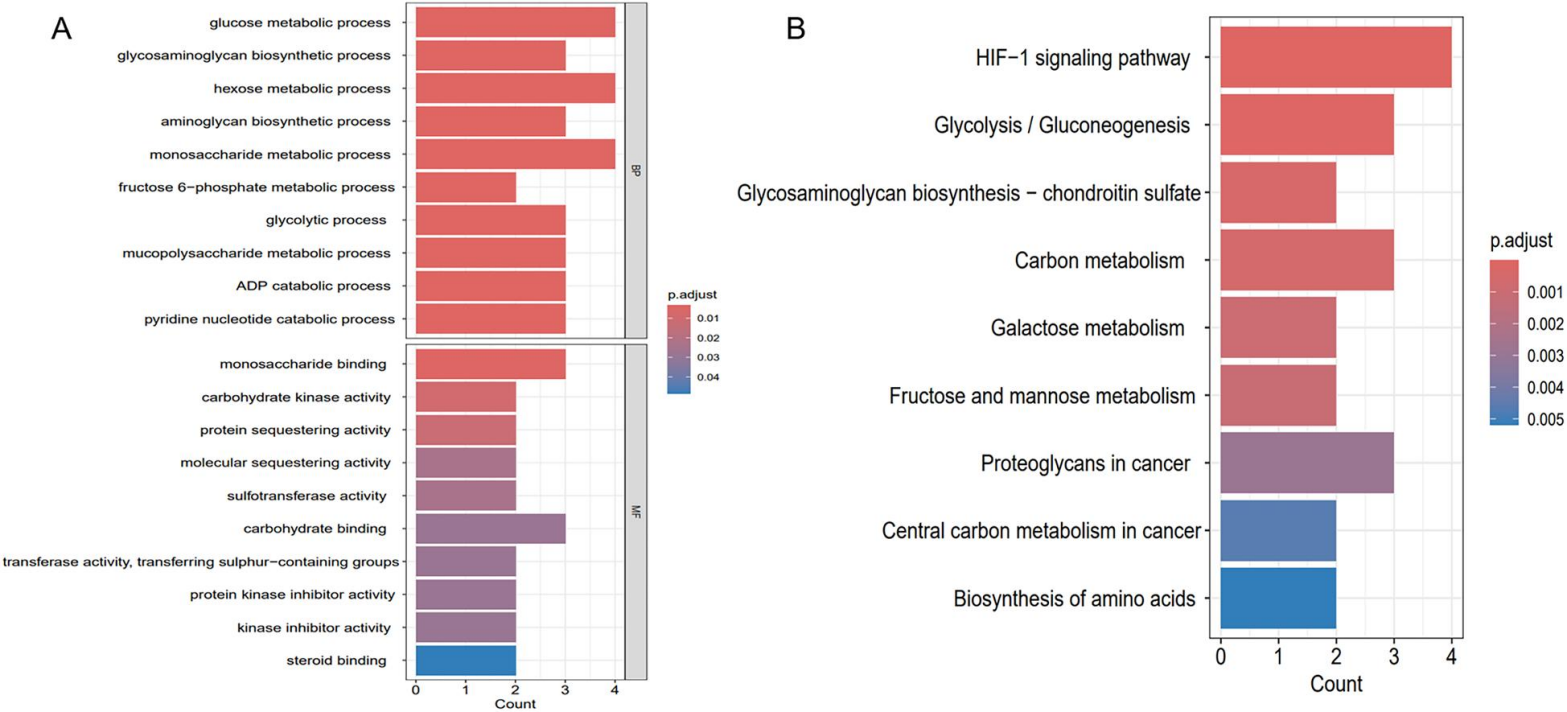
Enrichment analysis results. **(A)** GO enrichment analysis of key genes shared between hypoxia and AF. **(B)** KEGG enrichment analysis of key genes shared between hypoxia and AF.

To clarify the shared molecular biological processes of disease-linked genes, GO and KEGG enrichment analyses were carried out on the overlapping genes (Figure 6). The GO enrichment analysis highlighted the top 10 pathways linked to biological processes (BP) and molecular functions (MF), while the KEGG enrichment analysis identified the top 9 pathways. In the BP category, GO terms were predominantly linked to carbohydrate metabolism, ADP catabolic processes, and nucleotide metabolism. In the MF category, functional enrichment was observed in pathways related to monosaccharide binding and carbohydrate kinase activity. 9 pathways were identified after KEGG pathway enrichment analysis, revealing the clustering of hub genes in many pathways, including the HIF-1 signaling pathway, glycolysis/gluconeogenesis, glycosaminoglycan biosynthesis, as well as carbon metabolism.

### 3.4 Key genes selection via ML and evaluation of model diagnostic performance

**Figure 7:**
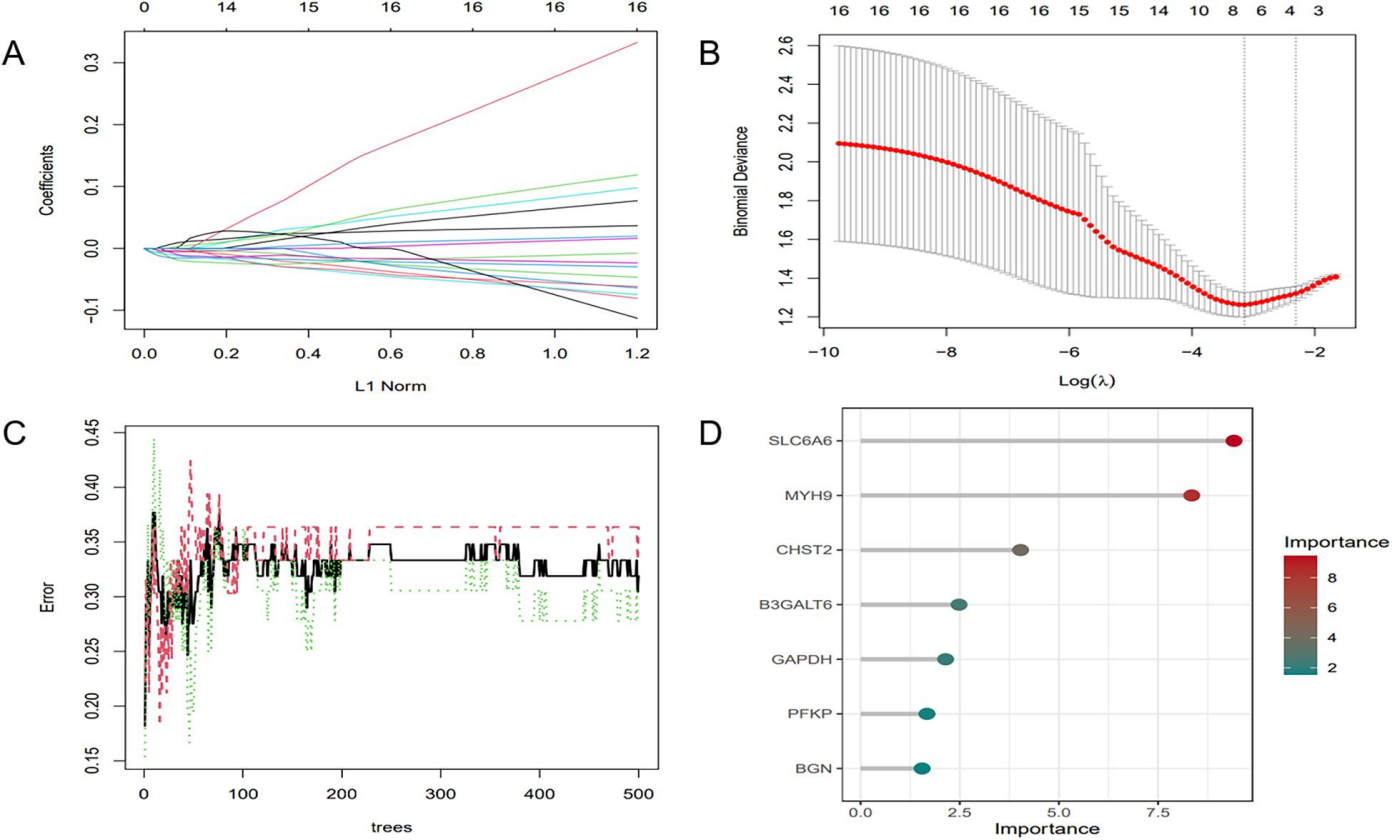
ML. **(A)** Regularization path of LASSO regression. **(B)** Cross-validation curve of LASSO regression. **(C)** RF model: the trend of error variation with the number of decision trees. **(D)** Feature selection results of the RF model.

**Figure 8:**
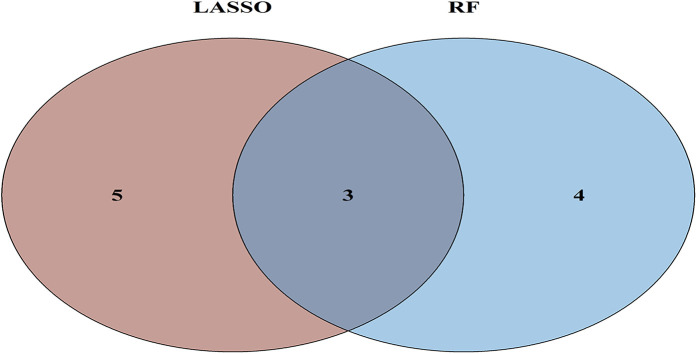
Venn diagram of ML results from RF and LASSO algorithms.

**Figure 9:**
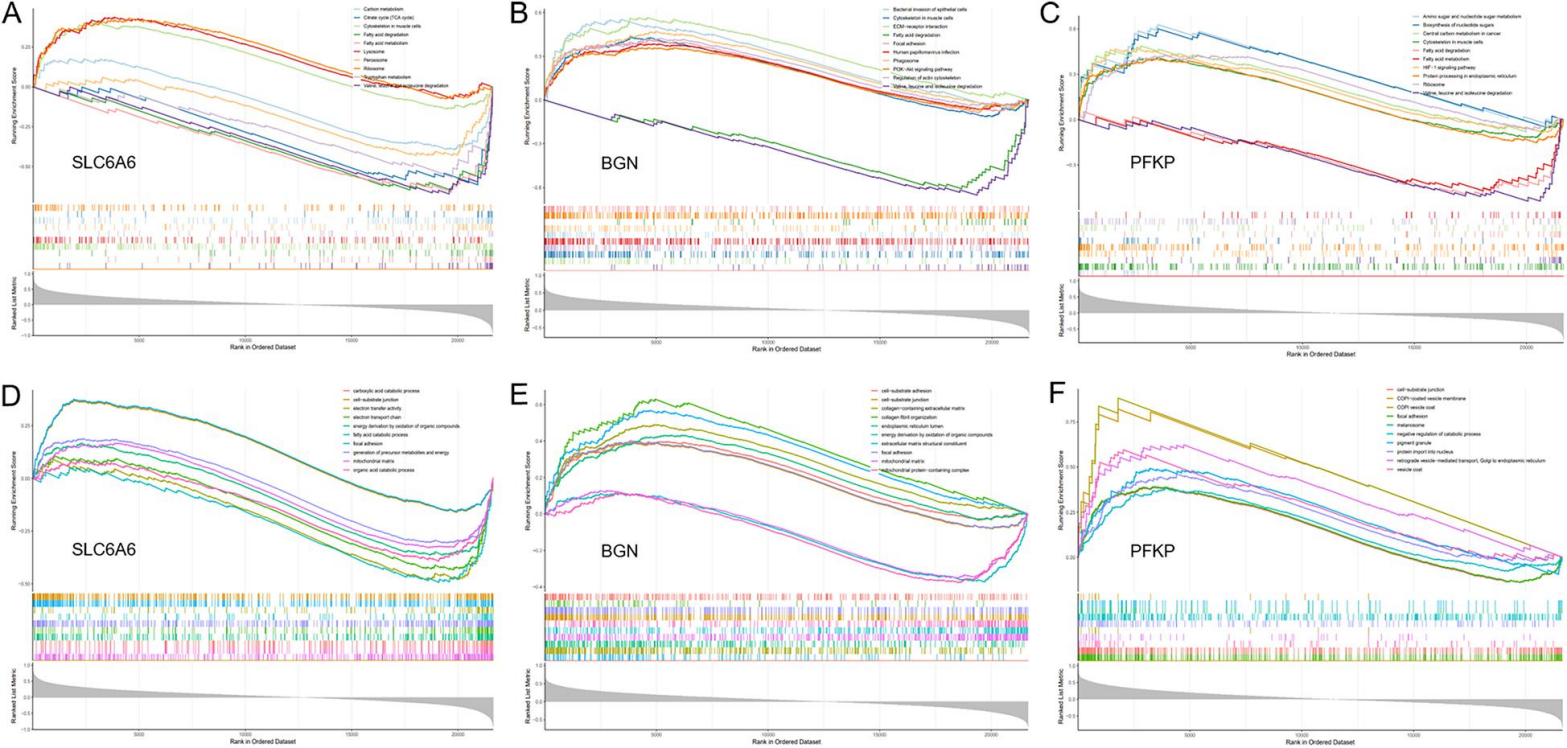
GSEA. **(A–C)** GO enrichment analysis of the key gene set (SLC6A6, PFKP, BGN) using GSEA. **(D–F)** KEGG enrichment analysis of the key gene set (SLC6A6, PFKP, BGN) using GSEA.

To further refine key gene selection, a 10-fold cross-validation analysis on the 16 overlapping genes was carried out via the LASSO algorithm (Figure 7B), and the optimal Lambda value (Lambda.min) was 0.04 (Figure 7A). Eight key genes were identified: HK1, PFKP, BGN, CDKN1A, CAV1, SLC6A6, P4HA1, and CHST. In the RF algorithm, the optimal tree number was 56, corresponding to the lowest error rate of 0.18 (Figure 7C). Genes with Mean Decrease Gini (MDG) scores > 2 were retained, corresponding to approximately the top 3% of features above the inflection point of the MDG distribution, thereby representing variables of high relative importance in the Random Forest model. Genes scoring over 2 were selected (Figure 7D), including SLC6A6, MYH9, CHST2, B3GALT6, GAPDH, PFKP, and BGN. Notably, SLC6A6, BGN, and PFKP were shared between LASSO and RF algorithms (Figure 8). To evaluate the predicting accuracy of critical genes for AF under hypoxic conditions, ROC curves were constructed, and the effectiveness was analyzed using metrics such as area under the curve (AUC), sensitivity, and specificity. The performance of the AF forecasting models built on RF and LASSO algorithms was examined (Figure 9). When candidate hub genes were used, the AUC values were as follows: SLC6A6, 0.736; BGN, 0.705; PFKP, 0.726. External validation using the GSE41177 dataset further corroborated the model's performance, yielding AUC values of SLC6A6, 0.891; BGN, 0.943; and PFKP, 0.953. Similarly, in the GSE79768 dataset, AUC values were 0.887 for SLC6A6, 0.613 for BGN, and 0.548 for PFKP. These findings collectively demonstrate robust predictive performance across datasets.

### 3.5 Differential expression of key genes and prognostic model development and evaluation

**Figure 10:**
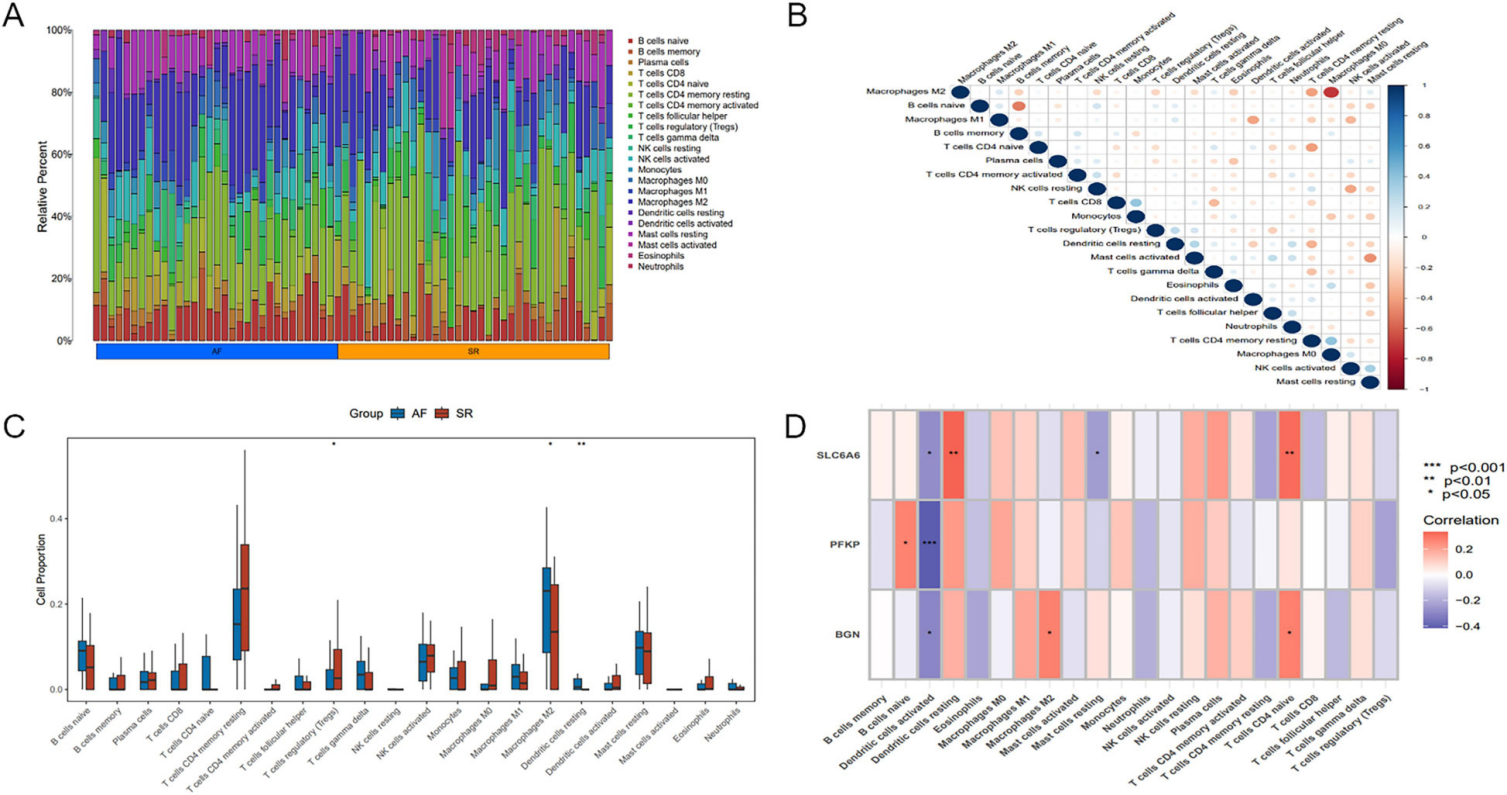
Immune infiltration analysis of key genes. **(A)** Heatmap illustrating differences in immune cell proportions between AF and SR samples. **(B)** Relationship among different immune cell types. **(C)** Boxplot of differences in immune cell proportions between AF and SR samples, blue represents AF patients, and red represents SR patients. **(D)** Correlation between the key gene set and immune cell populations. Statistical significance: *P < 0.05, **P < 0.01, ***P < 0.001.

The differential expression of key genes linked to AF and hypoxia was analyzed. Box plots (Figure 10A) showed that key genes exhibited markedly elevated expression levels in AF samples relative to SR samples (*P* < 0.05). Subsequently, a prognostic nomogram was constructed based on the expression levels of SLC6A6, BGN, and PFKP (Figure 10B) for risk assessment. The calculated score for each gene predicted the probability of AF occurrence. Calibration curves demonstrated minimal deviation between the observed and bias-corrected curves relative to the ideal curve (Figure 10C), indicating favorable predictive accuracy. Additionally, DCA demonstrated a significant net benefit (Figure 10D), highlighting the substantial clinical utility of the model in predicting AF during follow-up.

### 3.6 Immune cell infiltration analysis

**Figure 11:**
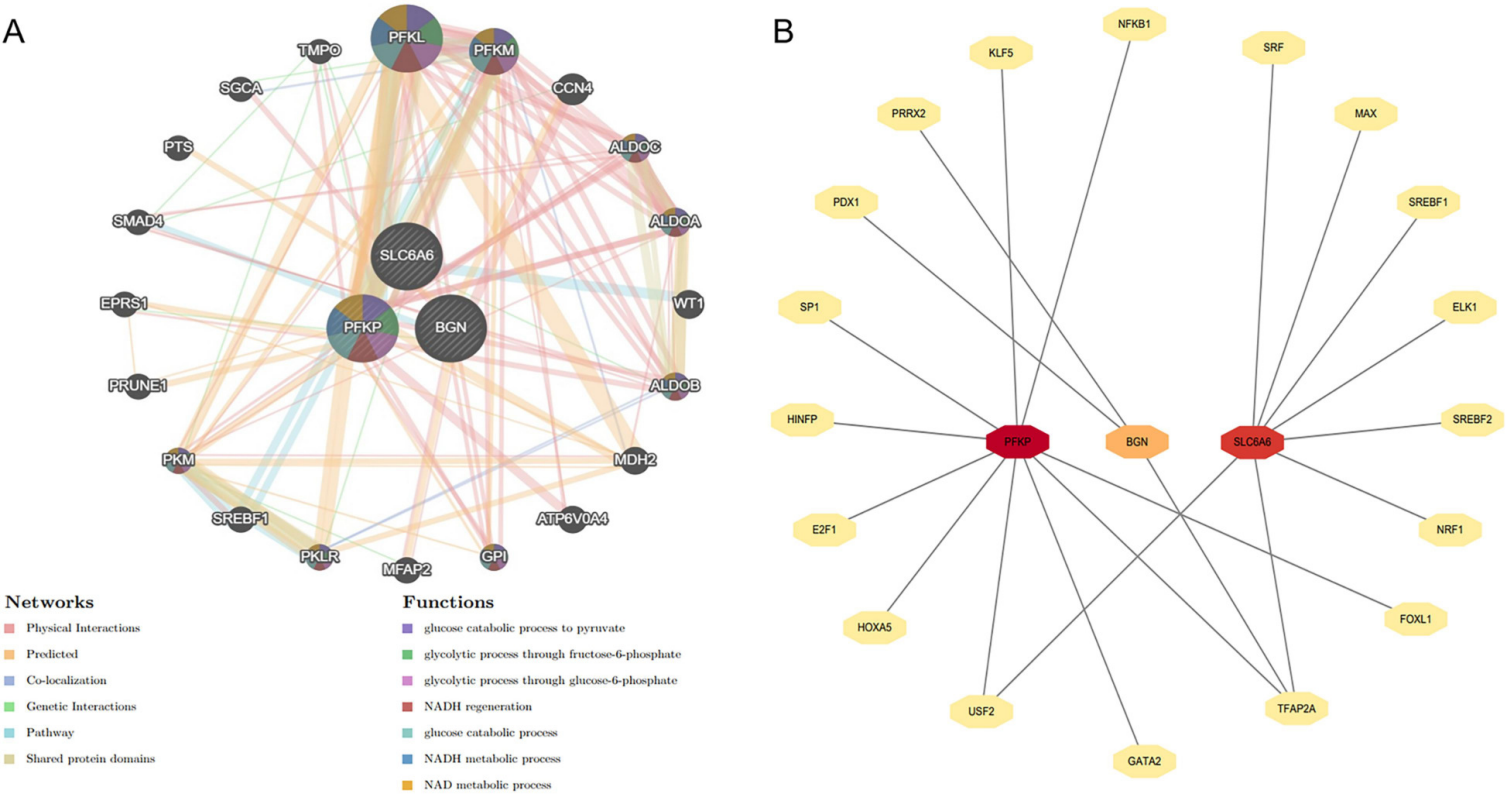
Construction of gene interaction network and prediction of upstream transcription factors. **(A)** The gene network analysis of the key gene set based on the GeneMANIA database. **(B)** Prediction of upstream transcription factors for the key gene set.

Significant differences existed in immune cell infiltration patterns across AF and SR samples (Figure 11A). Further comparative analysis of immune cell proportions (Figure 11C) demonstrated that the proportions of M2 macrophages (*P* < 0.05) and resting dendritic cells(DCs) (*P* < 0.01) were notably higher in AF samples, whereas the proportion of regulatory T cells (Tregs) (*P* < 0.05) was evidently higher in SR samples. Correlation analysis among different immune cell populations (Figure 11B) revealed strong negative or positive correlations, such as an inverse relation of M2 to M0 macrophages, and positive correlations of M2 with M1 macrophages as well as resting mast cells. Moreover, immune cell infiltration varied significantly among different key genes (Figure 11D). For instance, SLC6A6 expression was positively correlated with resting DCs and CD4+ T cells, PFKP was negatively correlated with activated DCs, and BGN was positively correlated with M2 macrophages but negatively correlated with activated DCs.

### 3.7 GSEA enrichment analysis and consensus clustering

**Figure 12:**
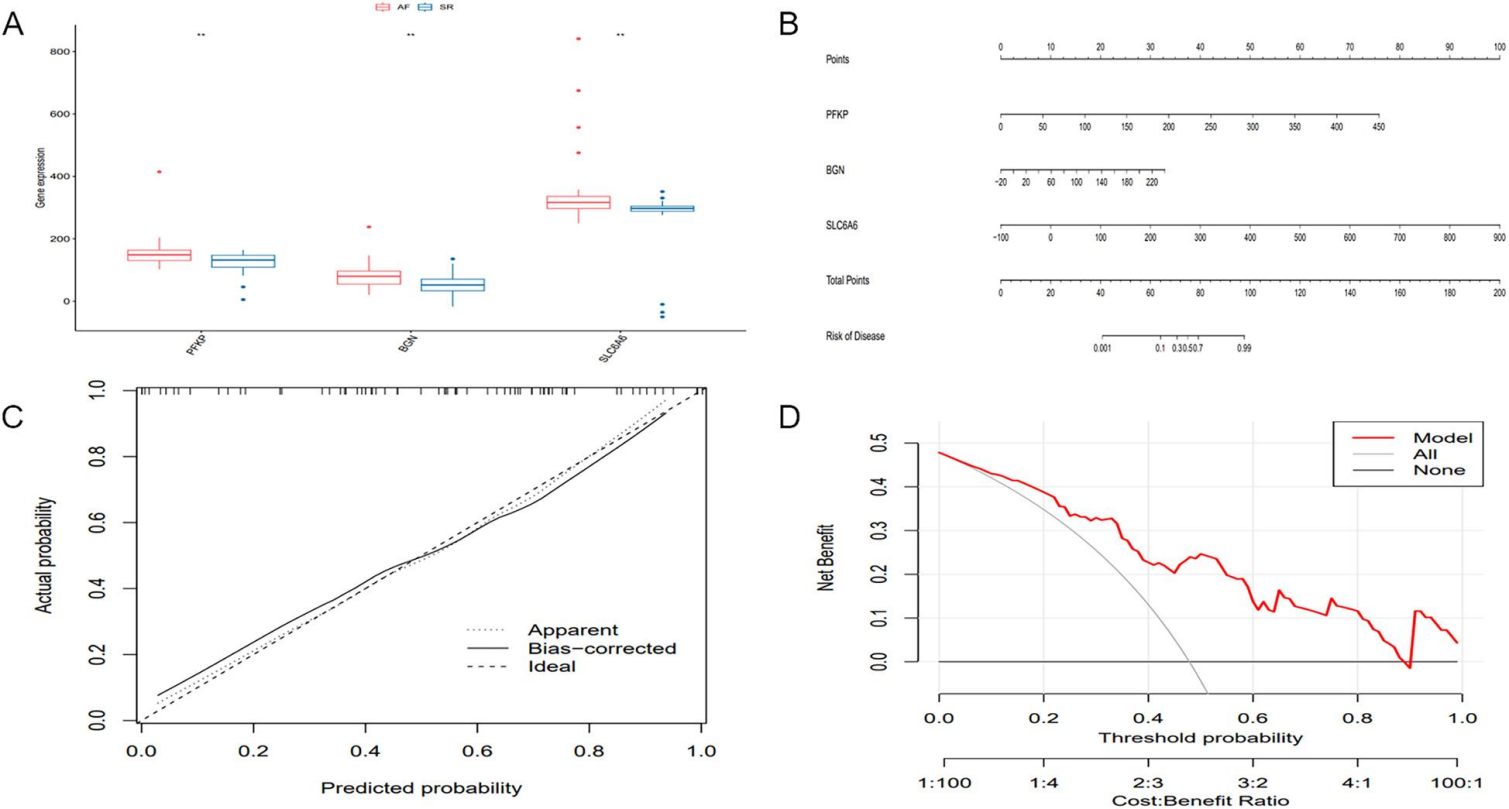
Differential expression of key genes and development and evaluation of the predictive model. **(A)** Boxplot of differential gene expression of the key gene set across different sample groups. **(B)** Nomogram model for predicting the probability of AF occurrence. **(C)** Calibration curve of the nomogram model. **(D)** DCA of the nomogram model.

**Figure 13:**
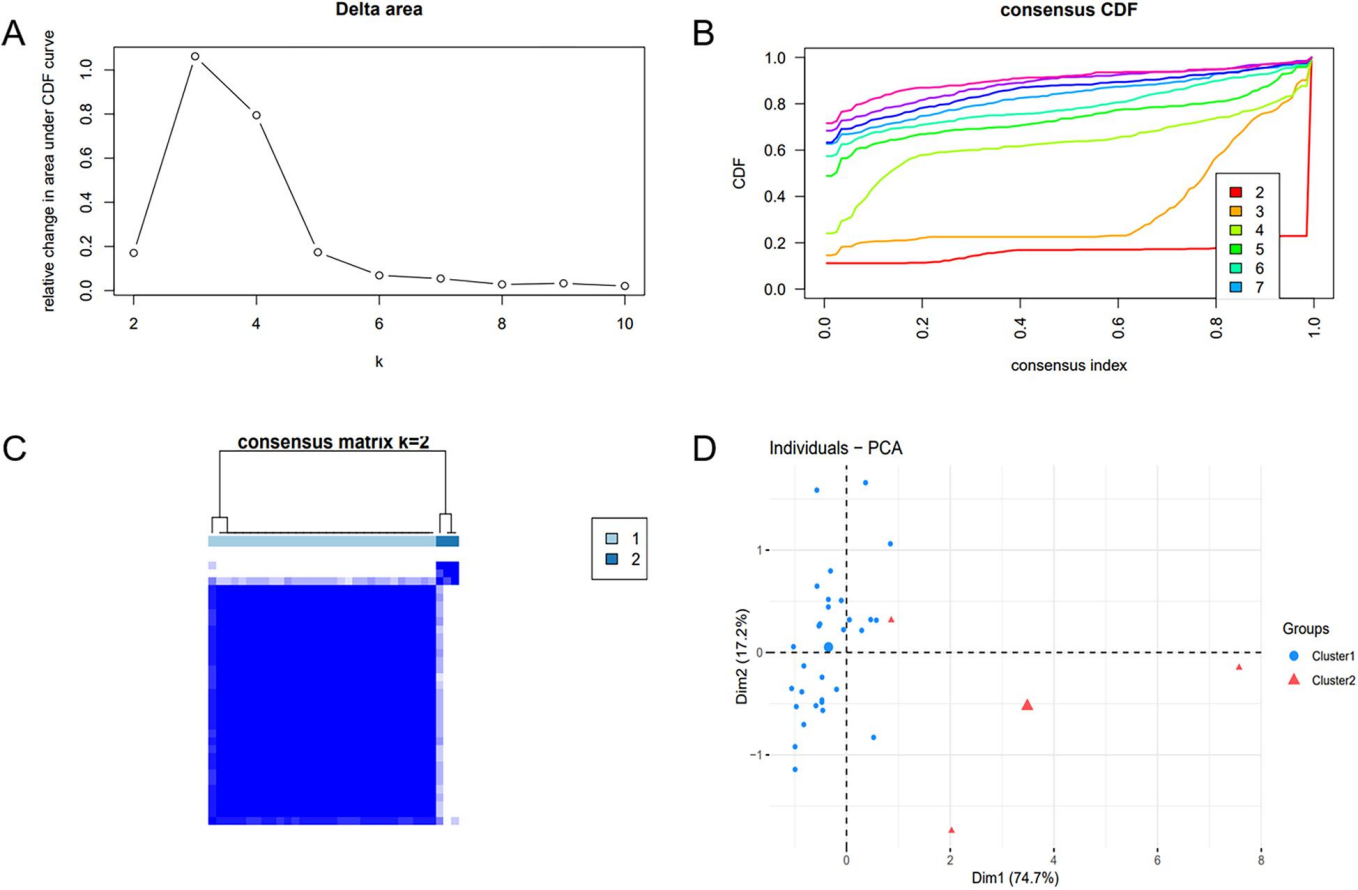
Consensus clustering analysis and PCA. **(A)** Delta area plot. **(B)** Cumulative distribution function (CDF) plot at *K* = 2. **(C)** Consensus matrix plot at *K* = 2. **(D)** PCA of AF samples.

To unveil the biological roles of key gene sets in AF, GSEA on three genes was carried out (Figure 12). The results revealed that the key genes (SLC6A6, BGN, and PFKP) are involved in distinct biological pathways, primarily including fatty acid and amino acid metabolism, interactions between cells and the extracellular matrix, and extracellular matrix biosynthesis. Furthermore, AF sample subtypes were classified through consensus clustering based on three genes. According to the cumulative distribution function (CDF) plot (Figure 13A) and Delta area plot (Figure 13B), heatmap analysis indicated that the optimal clustering of AF samples was into two groups (Figure 13C). PCA plot further illustrated the distribution of the two clusters (Figure 13D).

### 3.8 Construction of the gene interaction network and prediction of upstream transcription factors

**Figure 14:**
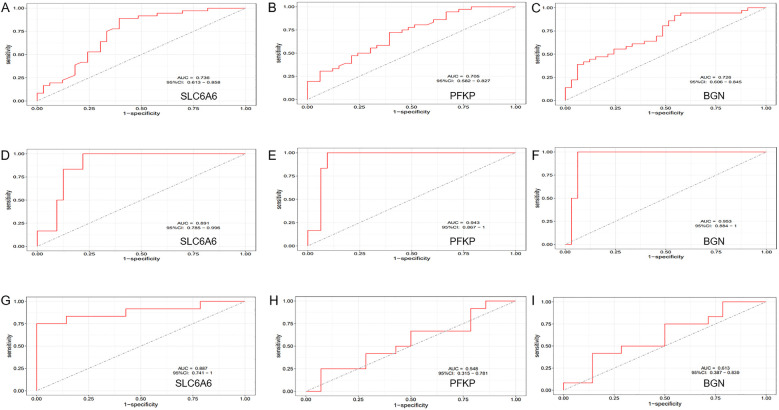
ROC Curve Analysis. **(A–C)** ROC curve analysis of the diagnostic efficiency of the key gene set (SLC6A6, PFKP, BGN) in the training dataset (GSE14975 combined with GSE115574). **(D–F)** ROC curve analysis of the diagnostic efficiency of the key gene set (SLC6A6, PFKP, BGN) in the external validation dataset (GSE41177). **(G–I)** ROC curve analysis of the diagnostic efficiency of the key gene set (SLC6A6, PFKP, BGN) in the external validation dataset (GSE79768).

The gene interaction network of key genes was formed using GeneMANIA (Figure 14A), providing insights into their potential roles in cellular functional regulation, transcriptional control, metabolic processes, and disease progression. The results demonstrated that this gene network is primarily involved in biological processes such as glycolysis and glucose catabolism. Moreover, upstream transcription factors of the key genes were forecast using the JASPAR transcription factor database (Figure 14B). Subsequently, Cytoscape 3.9.0 was employed to generate a related network diagram, illustrating the upstream transcription factors of key genes. The color intensity reflects the density of linked transcription factors.

## 4 Discussion

**Figure 15:**
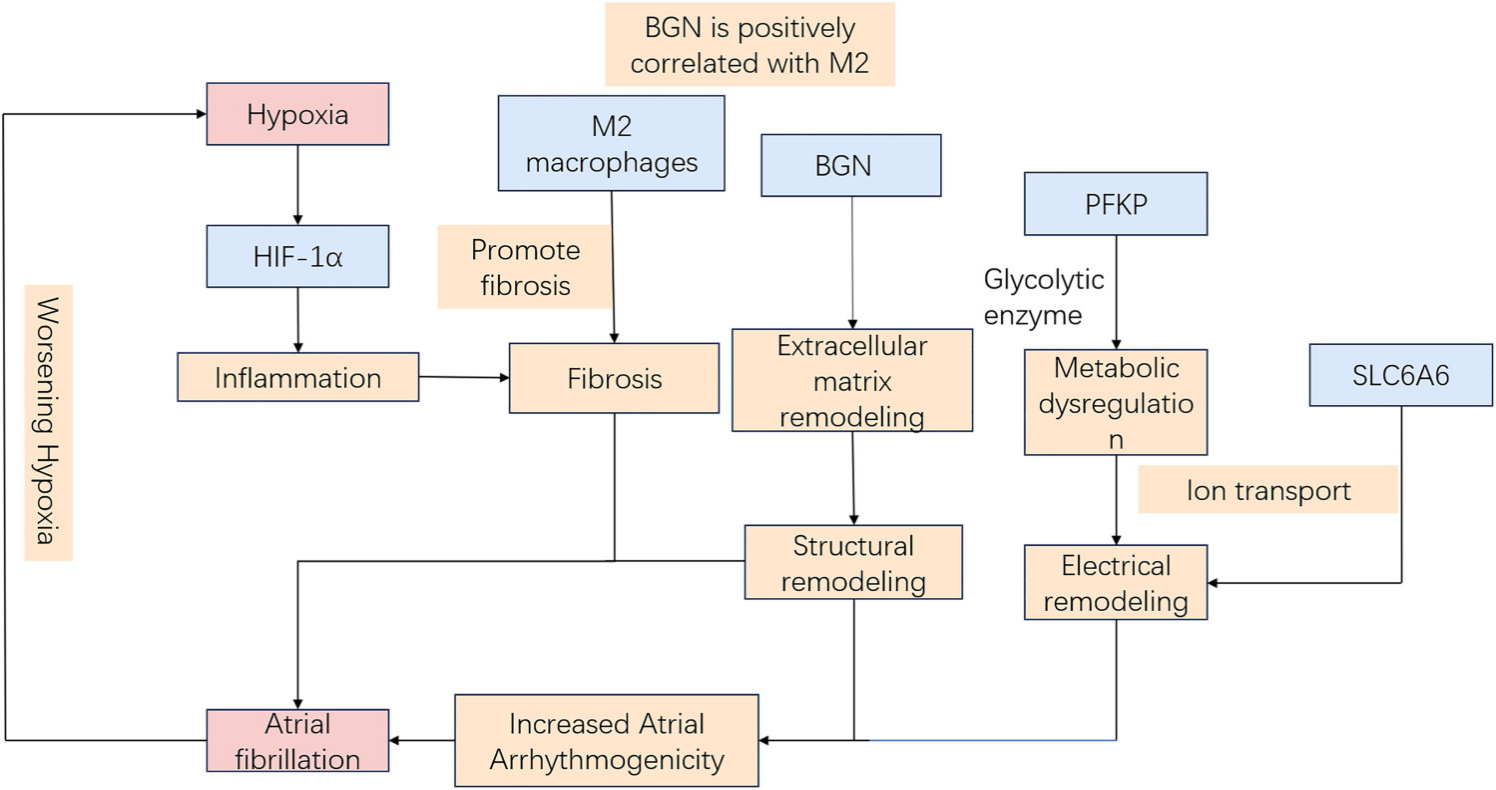
Schematic model of hypoxia-related mechanisms in AF.

Hypoxia induces electrophysiological changes in atrial cells, enhancing atrial excitability and susceptibility, thereby promoting AF. Oxidative stress and inflammatory responses further contribute to AF development by affecting cardiomyocyte function and electrical activity. Moreover, sympathetic activation, vagal responses, and atrial structural remodeling due to prolonged hypoxia [for instance, fibrosis (9)] play critical roles in AF pathogenesis. Hypoxia-inducible factor-1α (HIF-1α), a core molecular marker in hypoxia signaling pathways, has been implicated in AF onset and progression (10, 11), whereas studies on its downstream regulatory molecules remain limited. Therefore, identifying biomarkers related to hypoxia-induced AF is critical for diagnosing and treating this AF subtype. Our study leveraged WGCNA and ML approaches to identify three hypoxia-related key genes (SLC6A6, BGN, and PFKP). Based on these findings, a nomogram model was constructed to assess the diagnostic value of these key genes in predicting hypoxia-associated AF. Additionally, GSEA was conducted to elucidate their biological functions and specific involvement in biological pathways. Based on the current transcriptomic results and previous mechanistic evidence, it is proposed that hypoxia may promote AF pathogenesis through HIF-1α–mediated metabolic and extracellular remodeling pathways. As illustrated in Figure 15, SLC6A6, BGN, and PFKP occupy distinct yet convergent nodes within this regulatory network, potentially linking hypoxia-responsive signaling to atrial structural and electrophysiological alterations. Further experimental investigations are warranted to validate this hypothetical framework.

Enrichment analysis results indicate that the key genes all correlate with the HIF-1α signaling pathway. Studies have demonstrated that AF patients secondary to myocardial hypoxia exhibit elevated HIF-1α levels (12). Furthermore, HIF-1α may contribute to fibrotic remodeling, forming the pathological basis for AF induction (10). SLC6A6 primarily encodes a sodium-ion-dependent taurine transporter, which regulates cellular proliferation, differentiation, and apoptosis (13). Under hypoxic conditions, SLC6A6 is predominantly involved in energy metabolism-related activities. Existing research has shown that SLC6A6 is highly expressed in vascular smooth muscle cells (VSMCs), where its overexpression reduces reactive oxygen species (ROS) production and inhibits the Wnt/β-catenin pathway, thereby suppressing VSMC proliferation, migration, and dedifferentiation (14). Moreover, SLC6A6 overexpression further prevents vascular stenosis and atherosclerosis formation by inhibiting VSMC proliferation, dedifferentiation, and migration (15). Through its regulatory effects on cardiac energy metabolism and myocardial cell stability, SLC6A6 may indirectly participate in AF formation under hypoxic conditions. The BGN gene (Biglycan) encodes a glycosaminoglycan (GAG)-binding protein that primarily interacts with the extracellular matrix (ECM). BGN is expressed in multiple tissues, playing a crucial role in ECM structure and function. In this study, enrichment analysis revealed that BGN is mainly involved in ECM remodeling and energy metabolism-related biological processes, thereby promoting atrial fibrosis, electrical conduction heterogeneity, and oxidative stress, all of which contribute to AF development. The PREDICT-AF study, conducted by Nicoline et al., identified an association between BGN and AF, with elevated BGN expression observed in AF patients. The underlying mechanism is believed to involve fibroblast activation and interaction with collagen. During tissue remodeling in AF patients, increased BGN expression may serve as an early indicator of ECM remodeling in the atria (16). The PFKP gene encodes phosphofructokinase (PFK), a key enzyme in glycolysis that directly influences energy metabolism across various organs (17). In normal cardiac tissue, approximately 70% of energy supply is from fatty acid oxidation (FAO), while the remaining 30% originates primarily from glycolysis and the oxidation of lactate-derived pyruvate, which enters the mitochondria for oxidative phosphorylation (18). However, in the terminal stages of heart failure or under hypoxic conditions, the capacity for FAO is significantly damaged, leading to a marked shift toward increased glucose uptake and utilization (19). Consequently, PFKP is pivotal in the regulation of cardiac energy metabolism. Based on the enrichment analysis results in this study, PFKP is primarily involved in glycolysis-mediated energy supply and myocardial energy metabolism regulation. Aberrant PFKP expression or activity may result in insufficient ATP synthesis, affecting ion channel function, thereby altering myocardial electrophysiology, shortening the effective refractory period, and ultimately promoting AF development (20–22). A study by Marta et al. identified PFKP as a key factor in pathological cardiac hypertrophy (23). Additionally, PFKP is involved in the dynamic balance of focal adhesions, mediating cell-matrix adhesion via integrin regulation and potentially promoting collagen deposition and fibrosis through the TGF-β signaling pathway (24). Research has demonstrated that PFKP overexpression in proximal renal tubular epithelial cells exacerbates glycolysis and renal fibrosis triggered by TGF-β (25). Furthermore, Laurent et al. found that TGF-β1 induces PFKP expression, with a stronger induction observed in the pulmonary arteries of pulmonary arterial hypertension (PAH) individuals and cultured pulmonary arterial endothelial cells. This TGF-β1-induced PFKP expression can be inhibited by pioglitazone (26).

Furthermore, the key genes (SLC6A6, BGN, and PFKP) were positively linked to CD4+ T and B cells, and M2 macrophages and inversely related to DCs. Among the 22 immune cell types analyzed, M2 macrophages and resting DCs were notably elevated in AF samples, whereas Tregs were markedly reduced. Tregs are critical in preserving immunological tolerance and avoiding excessive immune responses. There was an evident reduction in the proportion of Tregs in patients with AF (27), possibly owing to impaired immune regulation and chronic inflammation, which may suppress cell proliferation during the pathogenesis of AF. The role of M2 macrophages in AF development has been well documented (28–30). Our immune infiltration analysis revealed a predisposition of M2 macrophages to infiltrate atrial tissue in AF patients. Upon activation by associated immune-inflammatory responses, this infiltration was accompanied by increased fibrotic area in cardiac tissue, enhanced collagen deposition, and upregulated fibroblast-to-myofibroblast transition, with concurrent activation of the TGF-β/Smad downstream signaling pathway, thereby further promoting fibrosis progression (30). DCs function as antigen-presenting cells and are essential for immune responses. However, how DCs contribute to AF pathogenesis remains unclear. Previous studies have indicated a marked rise in the proportion of immune cells in AF samples, with a significantly higher number of DCs in comparison to samples from individuals with SR (31, 32). In contrast, this study demonstrated a notable increase in the proportion of resting DCs in AF patients, whereas significant changes were not noted in activated DCs across AF and SR groups. Despite the high diagnostic accuracy of the hub genes identified through WGCNA and ML methodologies, which have been validated using external datasets, our study has limitations. First, the foregoing findings primarily rely on bioinformatics analyses of hub genes and *in vivo* and *in vitro* experimental validation is lacking. Therefore, conclusions regarding gene expression implicated in the molecular mechanisms of hypoxia-related AF should be interpreted with caution, warranting further experimental confirmation. Second, the possible influence of external clinical characteristics on the data were not accounted for. Additionally, limited genetic data were used in our immune cell infiltration analysis, and *in vivo* and *in vitro* studies were necessitated for unveiling the specific regulatory mechanisms.

### 4.1 Limitations

This study has limitations. First, Although the expression patterns and biological annotations of SLC6A6, BGN, and PFKP suggest that they may serve as molecular nodes integrating AF-related structural remodeling with hypoxia-responsive transcriptional networks, comprehensive experimental studies are necessary to confirm their causal roles in hypoxia-induced AF pathogenesis. Second, the external validation cohort (GSE41177) exhibited higher AUC values than the training cohort, likely due to the small control sample size and biological heterogeneity between datasets. Hence, these results should be interpreted as supportive rather than definitive evidence of model generalizability. Third, the immune cell infiltration analysis was performed using limited genetic data, and further *in vitro* and *in vivo* experiments are required to elucidate the underlying regulatory mechanisms. Lastly, the transcriptomic data were derived from left atrial tissue without direct measurement of oxygen tension, the identified hypoxia-related genes reflect molecular signatures of hypoxia responsiveness rather than confirmed evidence of actual tissue hypoxia.

## 5 Conclusion

In conclusion, our findings suggest that SLC6A6, BGN, and PFKP serve as potential hypoxia-related biomarkers and therapeutic targets in AF. Further investigations into immune responses may elucidate the molecular mechanisms underlying this condition and provide novel insights into the management of its comorbidities.

## Data Availability

The gene expression profiling datasets used in our study are publicly available in the Gene Expression Omnibus (GEO) database (https://www.ncbi.nlm.nih.gov/geo/) under the following accession numbers: GSE115574, GSE14975, GSE41177, and GSE79768.
